# Nonsyndromic Examples of Odontogenic Keratocysts: Presentation of Interesting Cases with a Literature Review

**DOI:** 10.1155/2019/9498202

**Published:** 2019-08-14

**Authors:** Naida Hadziabdic, Eda Dzinovic, Dalma Udovicic-Gagula, Nedim Sulejmanagic, Ahmed Osmanovic, Sabina Halilovic, Amina Kurtovic-Kozaric

**Affiliations:** ^1^Department of Oral Surgery, Faculty of Dental Medicine, University of Sarajevo, Bolnicka 4A, 71 000 Sarajevo, Bosnia and Herzegovina; ^2^Dental Polyclinic “Huskanovic”, Topolica 1, 75 000 Tuzla, Bosnia and Herzegovina; ^3^Department of Pathology, Clinical Center of the University of Sarajevo, Bolnicka 25, 71000 Sarajevo, Bosnia and Herzegovina; ^4^Private Dental Practice “Sulejmanagic”, Sarajevo, Avdage Sahinagica 11, 71 000 Sarajevo, Bosnia and Herzegovina; ^5^Department of Genetics and Bioengineering, Faculty of Engineering and Natural Sciences, International Burch University, Francuske revolucije bb, Ilidza, 71210 Sarajevo, Bosnia and Herzegovina

## Abstract

The odontogenic keratocyst (OKC) may occur at any age. However, it mostly occurs during the second and third decades of life. Compared to other odontogenic cysts, this type occurs with a frequency of 5-15%. It is more common in the mandible region and in the male sex. Histologically, odontogenic keratocysts are characterized by the presence of an external connective tissue capsule, with keratinizing lining of the epithelium consisting of 5-8 cell layers with marked palisadisation of polarized basal cells and a corrugated parakeratin layer. The objective of this study is to present cases of odontogenic keratocysts, with reference to the latest classification and dilemmas in therapeutic doctrine. This project was realized in the form of descriptive studies, specifically in a series of cases. A collection of four individual cases was found at the Department of Oral Surgery. Due to the proper approach towards diagnosis, adequate and detailed histopathological analysis, and suitable therapeutic procedures, all cases of odontogenic keratocysts were successfully treated without complications. Enucleation of OKC, with a regular follow-up, proved to be the effective therapeutic choice for the patients described in this paper. Only in the case of recurrence would we consider other therapeutic options, primarily enucleation in combination with Carnoy's solution.

## 1. Introduction

The odontogenic cysts are a heterogeneous group of lesions which are, according to the new WHO nomenclature, classified into odontogenic inflammatory cysts (such as periapical/radicular cysts and collateral inflammatory cysts) and odontogenic and nonodontogenic developmental cysts [1]. In contrast to other odontogenic cysts, most authors agree that the odontogenic keratocyst (OKC) is unique due to its features, that is, local aggressiveness and high rates of recurrence. It is also specific due to its histological characteristics. Compared to other odontogenic cysts, OKCs occur with a frequency of 5-10%, or according to some authors, 12-14% [2]. However, scientific data regarding the incidence of OKC are heterogeneous, which is in fact a reflection of irregularities in diagnosing and selection of samples in individual studies. For example, in some studies, a lack of distinction was made between orthokeratinized and parakeratinized lesions, even though these two entities exhibit different histopathological characteristics and are recognized as different entities by the new WHO classification [3].

According to the literature, OKC is more common in the mandible, in the lateral region, with the highest incidence in patients being between ten and thirty years of age [2]. It shows mild predominance among male patients [4], whilst taking into account that there is no significant inclination of these lesions with regard to gender [2, 5]. The research by Chirapathomsakul et al. has revealed that the OKC male to female manifestation ratio is 1.6 : 1 [6]. Keratocysts are usually detected accidentally during regular radiographic imaging. The same lesion is represented as a unilocular or multilocular radiolucent formation with a sclerotic border towards the surrounding bone [7]. Although there are cases without symptoms, a number of authors emphasize that clinical manifestations, such as swelling and pain, may occur, individually or in combination [4]. Histologically, OKC is characteristically composed of uninflamed fibrous walls, lined by a stratified squamous epithelium, which is 5-8 layers thick with a palisaded hyperchromatic basal cell layer and “corrugated” parakeratotic epithelial cells on the luminal surface [1, 6, 8].

From 1950 to 2017, the classification of odontogenic keratocysts (OKCs) underwent substantial changes. Chronologically, the name “keratocyst” was first introduced in 1950. The same name was officially used in the WHO classification from 1971 to 1992. According to these classifications, there were two subtypes of odontogenic keratocyst (OKC): the parakeratinized and orthokeratinized types. However, in 2005, these two types were classified as independent entities, mostly because of the differences in their tendency to recur. The parakeratinized type was classified as a “keratocystic odontogenic tumor” (KCOT) under the branch of epithelial tumors of odontogenic origin. The orthokeratinized type was classified as an orthokeratinized odontogenic cyst (OOC) as part of the entity of odontogenic developmental cysts. According to the newest WHO classification from 2017, keratocystic odontogenic tumors (KCOT) were again reclassified as odontogenic keratocysts (OKCs) but still diagnostically distinctive from orthokeratinized odontogenic cysts (OOC) [9, 10].

One of the reasons for the WHO's reintroduction of the term “odontogenic keratocyst” (OKC) was because *PTCH1* gene mutations were found in other developmental cysts [1, 11]. Another reason was the fact that marsupialization is an effective treatment method for OKC and may be associated with the return of the epithelium to normal, with lower rates of recurrence, which is not a characteristic of neoplasms [3, 5, 12]. It should also be said that OKCs are one of the diagnostic criteria for nevoid basal cell carcinoma syndrome, that is, Gorlin Goltz syndrome, inherited in an autosomal dominant fashion with variable expression [13–16].

The origin of OKC is not quite clear. It is presumed to be either primordial, including dental lamina remnants, basal cells of the overlying epithelium, or dentigerous, which implies reduced enamel epithelium of the dental follicle [17].

In this paper, the authors present 4 interesting cases from their own practice. The cases described are particularly interesting for their clinical characteristics (localization, symptoms) and differential diagnosis. The paper also contains a mini literary review, in which the authors refer to the latest classification of odontogenic keratocysts and their therapeutic possibilities.

## 2. Clinical Cases (from Our Own Experience)

### 2.1. Case 1: Keratocyst in the Region of the Maxillary Left Canine (Figures 1(a)–1(d))

#### 2.1.1. Anamnesis and Clinical Overview

**Figure 1:**
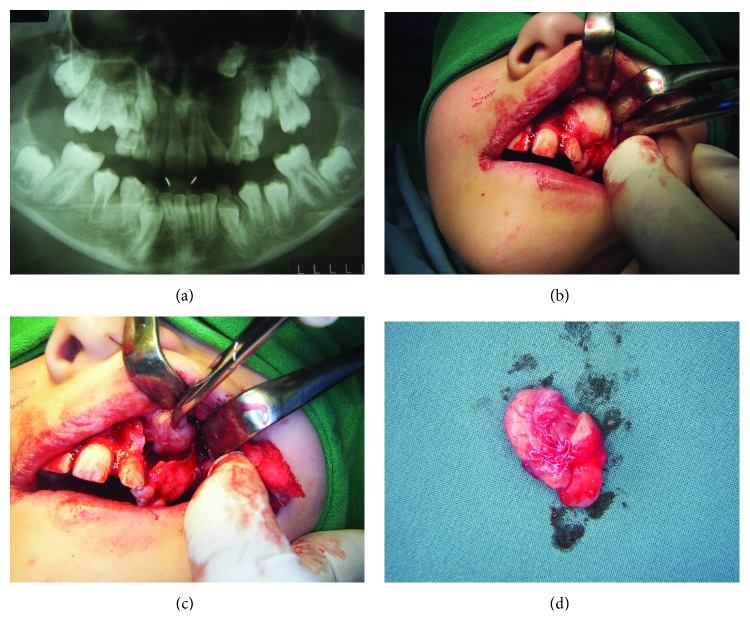
(a) Ellipsoid RTG radiolucency in the left maxilla which diverges the roots of adjacent teeth, in the upper part of which there is a retained permanent canine. (b) Opalescent-white cyst sheath after the mucoperiosteal flap has been raised. (c) Enucleation of a cystic capsule. (d) Macroscopic preparation of exterminated keratocysts together with a retained canine tooth.

A 10-year-old patient arrived at our clinic due to a tumefaction in the frontal region of the maxilla, especially above the deciduous canine on the left. Clinical examination revealed deformity of the maxilla in that region, in the form of a round swelling. The bone in the described area had been reduced to egg-shell thickness, and a crackling sensation was felt on pressure (Dypitren's phenomenon). There was no pain. Inspection also revealed the presence of an ectopic lateral permanent incisor on the left (Figure 1(b)).

#### 2.1.2. X-Ray Findings

On the orthopantomogram (OPG) image, ellipsoidal, longitudinally positioned radiolucency was observed, which diverged at the roots of the adjacent teeth, i.e., the lateral incisor and the deciduous canine. In the apical part of this radiolucency, the crown of the retained permanent canine was prominent (Figure 1(a)). According to these clinical characteristics and X-ray analysis, this was assumed to be a dentigerous cyst of the maxilla with the retained canine.

#### 2.1.3. Surgical Report

Under local anesthesia, a trapezoidal flap was raised. Subsequently, it was noticed that the cyst had completely expelled the bone fragment from the vestibular side as it grew. The thinned, vestibular bony wall was carefully cut off and removed. Thus, the boundaries of the cyst, containing a protective white outer shell, were displayed (Figure 1(c)). The cyst was completely removed along with the associated retained tooth (Figure 1(d)). The wound was irrigated and sutured, allowing healing by primary intention. The patient was prescribed the appropriate antibiotic, analgesic, and anti-inflammatory therapy. No complications were noted in the postoperative period except that the swelling remained a day longer after the operation, but there was no pain or signs of inflammation. Due to the large bone defect, which was the result of the growth of the cyst, temporary orthodontic therapy was delayed in order to await bone regeneration.

#### 2.1.4. Pathology

**Figure 2:**
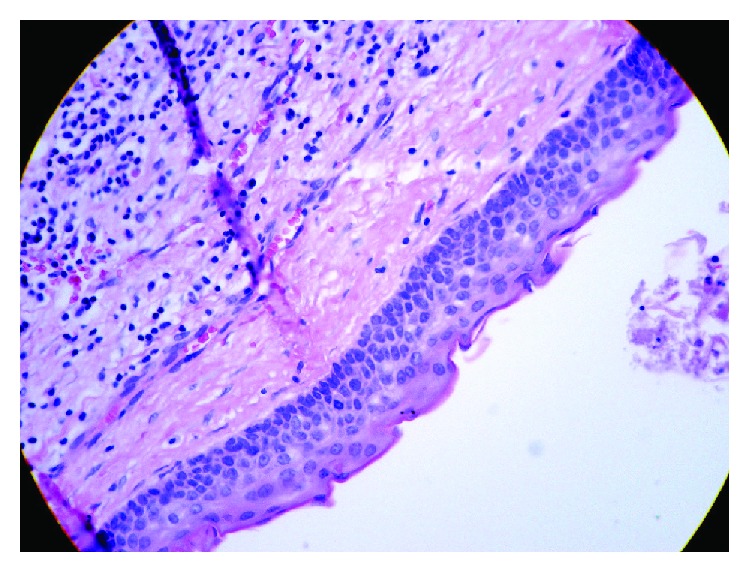
The characteristic corrugated surface of the epithelium, with a polarized layer of palisaded basal cells, and keratin stripped of the HE ×40.

Histopathological analysis of the removed cyst revealed characteristics of odontogenic keratocysts rather than dentigerous cysts as originally believed on clinical examination and radiographic diagnostics. The histopathological results had also showed the characteristic corrugated lining of parakeratotic epithelial cells on the luminal surface of the cyst wall, with a palisaded hyperchromatic basal cell layer supporting and strengthening the fibrous wall of the cyst (Figure 2).

#### 2.1.5. Follow-Up Period

**Figure 3:**
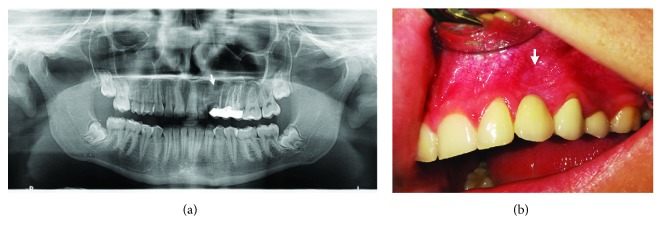
Follow-up period after 9 years. (a) An orthopantomogram (OPG) with no signs of radiolucency with satisfactory bone regeneration in the operated area (arrow). (b) Clinical appearance of vestibular mucosa with the presence of mild recess (arrow).

The follow-up period is 9 years. The patient has no symptoms and no clinical or radiological signs of recurrence (Figure 3).

### 2.2. Case 2: Keratocyst in the Region of the Upper Left Third Molar (Figures 4(a)–4(c))

#### 2.2.1. Anamnesis and Clinical Overview

The patient came to the Oral Surgery Department for the surgical removal of an upper impacted third molar on the left. The patient stated that she had had swelling in the region several times and she had been repeatedly under antibiotic therapy.

#### 2.2.2. X-Ray Findings

**Figure 4:**
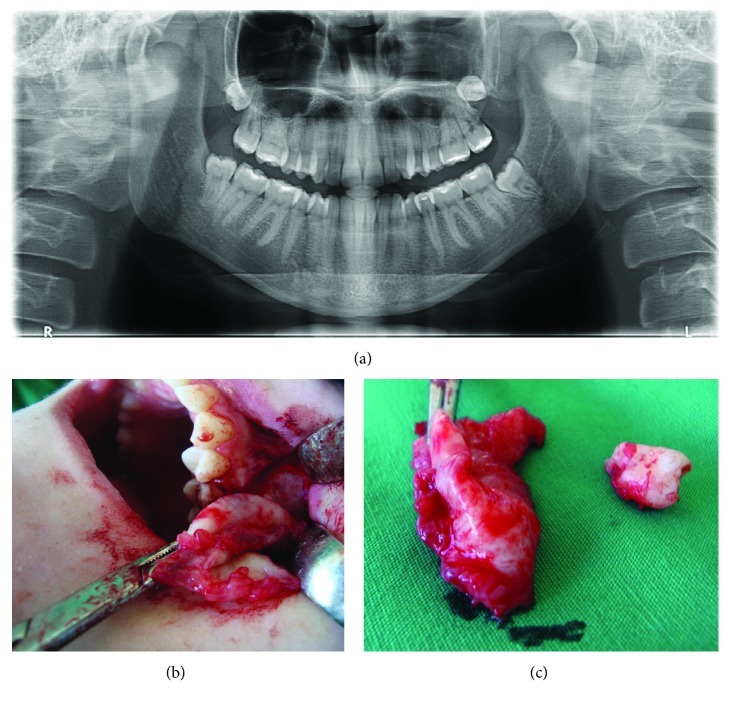
(a) OPG image showing a highly impacted 28 with opacification of the left maxillary sinus. (b) Enucleation of a huge cystic capsule. (c) Macroscopic preparation of a cystic capsule and extracted impacted tooth.

The crown of the impacted tooth 28 was located above the root of tooth 27 with obvious radio-opacity of the left maxillary sinus when compared to the right side (Figure 4(a)).

#### 2.2.3. Surgical Report

After application of local anesthesia, a triangular flap was raised and the bone exposed. After corticotomy of the vestibular side above the apex of tooth 27, the crown of tooth 28 was shown. During the extraction of tooth 28, there was a leak of thick, dirty yellow content that filled a large cystic lesion. The cyst was carefully enucleated (Figure 4(b)). Its dimensions were about 5 × 8 cm (Figure 4(c)). No relation was found between the maxillary sinus antrum and the bone defect. The wound was irrigated thoroughly and sutured. Ampicillin antibiotic, in combination with metronidazole, was prescribed as therapy. At the first postoperative follow-up appointment, an edema with moderate hematoma of the cheeks was noticed, but without pain. The further healing has proceeded smoothly.

#### 2.2.4. Pathology

**Figure 5:**
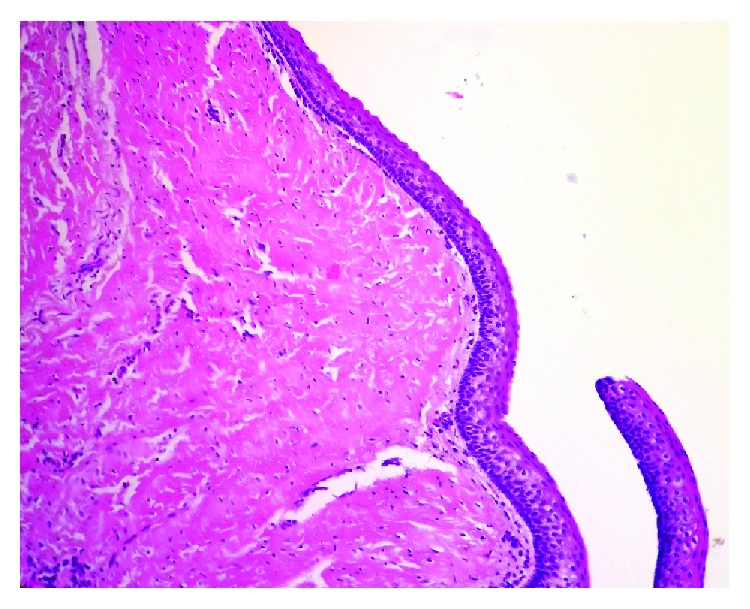
The cystic fibrous wall is coated with a thin stratified epithelium with an accumulated parakeratotic layer on the surface. The basal layer of the cells shows characteristic palisadisation of the hyperchromatic nuclei.

The results of histopathological analysis indicated that the site was covered with a multilayered, parakeratotic, squamous stratified, lining epithelium with a preserved basal cell layer. Infrequent inflammatory infiltrates, rich in lymphocytes and plasma cells, could be detected in the subepithelial parts (Figure 5). Histopathological diagnosis supported odontogenic keratocysts.

#### 2.2.5. Follow-Up Period

**Figure 6:**
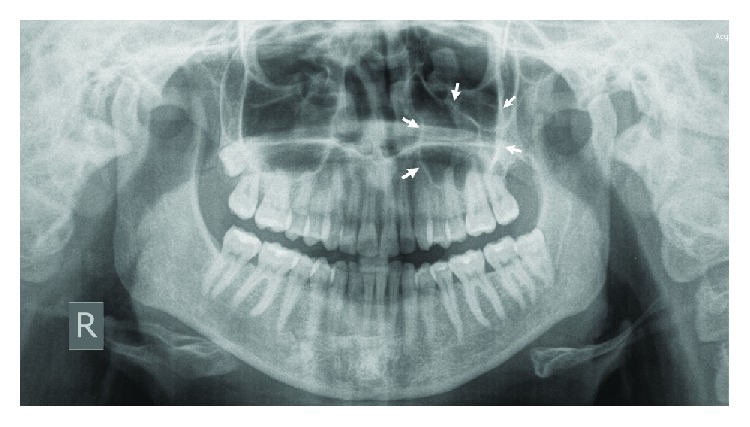
Control OPG with no radiological signs of recurrence.

The follow-up period is 6.5 years with no radiological signs of recurrence (arrows indicate the area of interest) (Figure 6).

### 2.3. Case 3: Large Odontogenic Keratocyst in the Mandible (Figures 7(a)–7(d))

#### 2.3.1. Anamnesis and Clinical Overview

**Figure 7:**
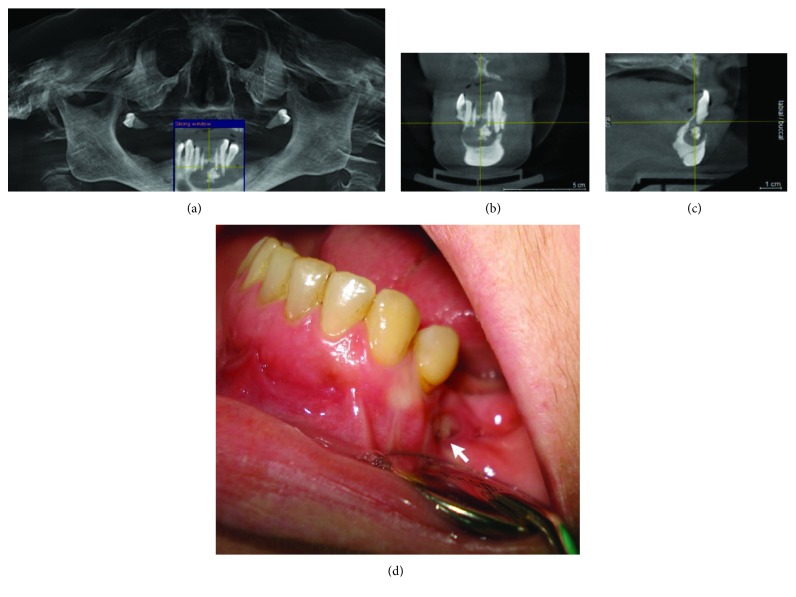
CBCT image: (a) panoramic view, (b) axial view, and (c) cross-sectional view. (d) Fenestration on the mucous membrane.

**Figure 8:**
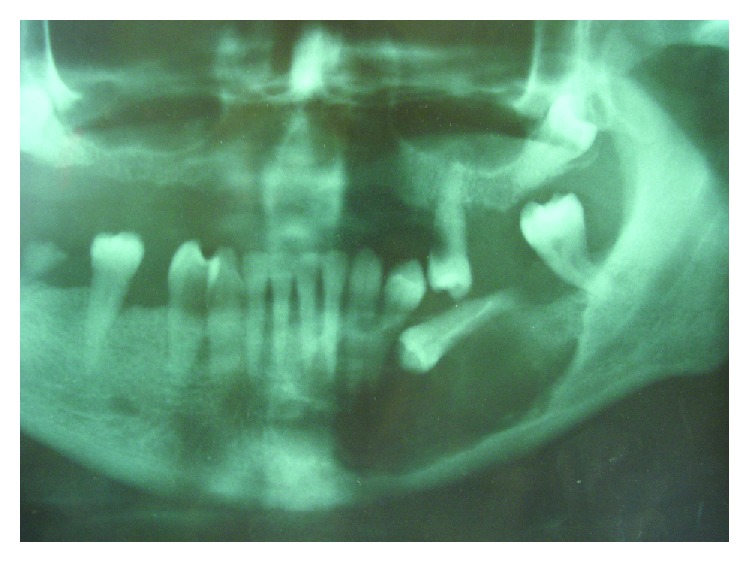
A detail from the OPG showing an impacted 35 and clearly lined RTG radiolucency in the region of 33-37 when compared to the other parts of the mandible.

The patient came to the Department of Oral Surgery at the Faculty of Dental Medicine, University of Sarajevo, for fenestration of vestibular mucosa in the tooth 34 region and edema and mandible pain in region 34-43. Anamnesis revealed the outpatient removal of a semi-impacted tooth 35 years ago (Figure 8). Clinical examination showed a fistula opening in region 34 (Figure 7(d)) of about 0.5 × 0.5 cm. A milder edema in the vestibulum in the 34-43 region was noticed that fluctuated in the region of teeth 41 and 31. During palpation, the patient complained of moderate pain. All 34-44 teeth were intact. Teeth 34-42 showed a moderate degree of mobility. The patient denied trauma as a possible etiological factor.

#### 2.3.2. X-Ray Findings

CBCT analyses (Figures 7(a)–7(c)) showed well-defined radiolucency in the 34-43 area that matched the cystic lesion. When irrigating the cystic cavity through the fistula tract with sodium hypochlorite, large amounts of dirty contents mixed with food remains were observed. Prior to surgical treatment, the patient was repeatedly called for control examinations during which abundant irrigation with NaOCl was undertaken.

#### 2.3.3. Surgical Report

**Figure 9:**
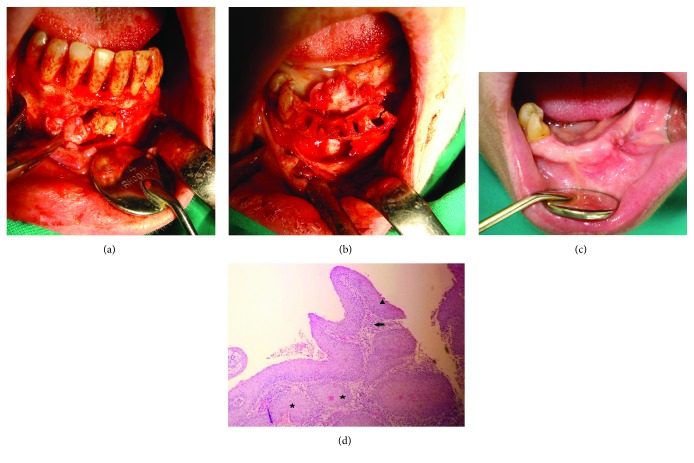
(a) Procedure for removal of the cystic wall lining and contents. (b) Cavity in the bone after removal of the cyst and extraction of six teeth. (c) Clinical appearance 2 months after surgery. (d) Inflamed fibrous wall lined with nonspecific stratified squamous epithelium (arrow) with focally corrugated surface (arrowhead). Satellite cysts and solid islands in the wall (star).

After application of local anesthesia (V anesthesia and left mandibular block), a trapezoidal flap in the region of 34-43 was raised. It was noted that the lesion had destroyed the vestibular bone in regions 31 and 41 (Figures 9(a) and 9(b)). The existing bone defect was extended in order to make a wider osseous window to facilitate enucleation of the cyst. During enucleation, a hard formation of about 1 cm in size, which appeared similar to a bony sequestrum, was removed. At the patient's request, teeth 34, 33, 32, 31, 41, and 42 were extracted. The sharp bone edges were flattened. Excision of the affected mucosa was performed. The wound was irrigated; the mucoperiosteal flap deperiostated and sutured. The surgical procedure went well. Antibiotics, anti-inflammatory drugs, and analgesics were prescribed in therapy. The first checkup, three days post operation, went smoothly. Further therapy was potentiated by procedures to reduce the edema and accelerate epithelialization (Figure 9(c)).

#### 2.3.4. Pathology

The microscopic finding showed that on the surface of the sample there was a uniform multilayer epithelium without the presence of rete ridges. The basal cell layer was characterized by remarkable hyperchromatic nuclei. The luminal surface was corrugated and parakeratotic. In the cystic wall, there were epithelial cell invaginations that had produced satellite cysts and chronic inflammatory infiltrates. Histopathological analysis confirmed the diagnosis of odontogenic keratocyst (Figure 9(d)).

#### 2.3.5. Follow-Up Period

Two years after the second operation, the patient did not follow through with regular checkups so data about possible recurrence are not available.

### 2.4. Case 4: Large Odontogenic Keratocyst in the Maxilla (Figures 10(a)–10(d))

#### 2.4.1. Anamnesis and Clinical Examination

**Figure 10:**
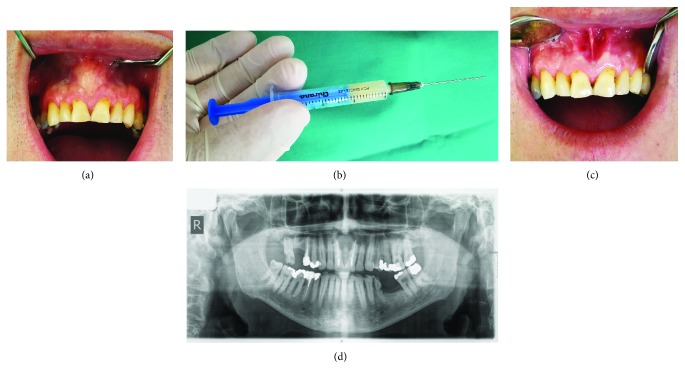
(a) Tumefaction of the upper vestibule above the central incisors. (b) Cystic aspirate. (c) Dent in the vestibular mucosa after the aspiration of the cystic content. (d) OPG image with a sharp shadow diverging the roots of teeth 11 and 21.

The patient visited the Oral Surgery Department due to a swelling in the frontal vestibular region of the upper jaw above the central incisors (Figure 10(a)). A clinical examination established a visible tumefaction of that region that fluctuated during palpation. Probation puncture showed a light yellow, blurred content (Figures 10(b) and 10(c)). From the anamnesis, it became evident that the patient had received a blow to the frontal part of the upper jaw several years before. Therefore, in terms of differential diagnosis, it was suspected to be a radicular cyst of the maxilla with trauma as an etiological factor. For this reason, trepanation and endodontic treatment of teeth 11 and 21 was indicated and this was successfully undertaken.

#### 2.4.2. X-Ray Findings

A large radiolucency that diverged at roots 11 and 21 was visible on the orthopantomogram (OPG) X-ray (Figure 10(d)).

#### 2.4.3. Surgical Report

**Figure 11:**
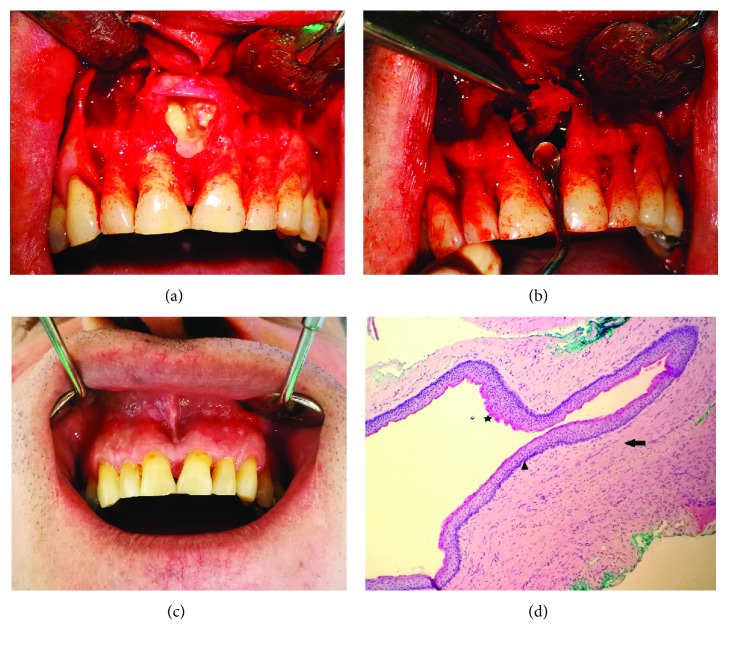
(a) Destruction of the vestibular lining of the maxillary bone through which the thick cystic content can be seen. (b) Enucleation of a cystic capsule. (c) Clinical appearance of the upper vestibule a month after surgery. (d) Typical histology of an odontogenic keratocyst (OKC). Uninflamed fibrous wall lined by a thin regular parakeratinized epithelium, 5-8 cell layers thick (arrow). The parakeratinized surface is corrugated (star) and the basal layer is palisaded with hyperchromatic nuclei (arrowhead).

Following the application of local anesthesia, a trapezoidal flap was raised. After that, it was observed that the vestibular part of the bone had been destroyed along the width of the lesion (2.5 × 2.0 cm). A noticeable cavity in the bone was filled with dense yellow content (Figure 11(a)). After the removal of these contents, the cyst sack was carefully enucleated (Figure 11(b)). Subsequent to the removal of the cystic parts, it was noted that part of the bone was missing from the palatal side, as well as the bone bound to the nose. The nasal mucous membrane remained intact. The wound was irrigated and sutured. After a month, the wound had healed completely (Figure 11(c)).

#### 2.4.4. Pathology

Histopathological analysis showed the characteristic histological image of a keratocystic odontogenic tumor with a multilayer epithelium of 5-8 cell thickness and a corrugated and parakeratinized surface, with the absence of inflammatory cell infiltrates in the underlying supportive connective tissue (Figure 11(d)).

#### 2.4.5. Follow-Up Period

**Figure 12:**
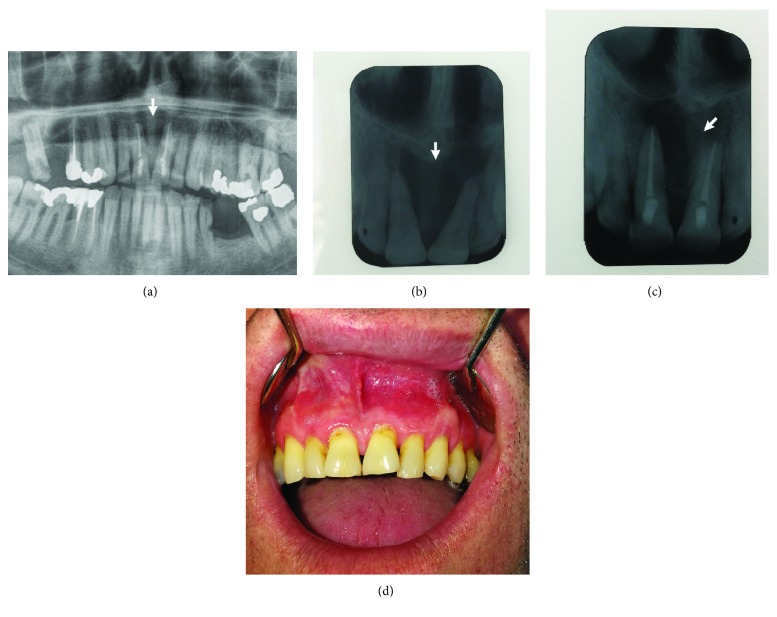
Follow-up period one 1 year after surgery. (a) OPG with no signs of radiolucency. (b) Preoperative X-ray with obvious signs of radiolucency. (c) Postoperative X-ray one year after surgery with signs of bone regeneration in progress. (d) Clinical appearance of vestibular mucosa one year after surgery (no swelling, no inflammation).

The follow-up period is 1 year. The patient is symptomless with adequate signs of bone regeneration evident on X-ray analysis (Figures 12(a)–12(d)).

## 3. Discussion

The classification of odontogenic keratocysts (OKCs) is still a topic commonly discussed by many dental researchers. According to the most recent WHO classification, the preferred term remains odontogenic keratocysts due to the lack of evidence of its neoplastic nature [18]. This observation has caused the disapproval of some scientists, such as Stoelinga, who called this decision “somewhat strange, denying its behavior as a benign but aggressive tumor” [19]. What was definitely clarified in the newest WHO classification is that OKC should be distinguished from orthokeratinized odontogenic cysts (OOC), which represent a separate entity of developmental cysts with clearly defined histological characteristics: a thin epithelium (4-9 cell layers thick, with signs of orthokeratosis) [20, 21]. On the other hand, a typical odontogenic keratocyst shows characteristic corrugated parakeratotic epithelial cells on the surface and hyperchromatic palisaded basal cells [20, 21]. For clinicians, it is important to relate OKCs to their high recurrence rate (especially syndromic-related lesions and multilocular lesions) [18]. OOC has a low recurrence tendency [22, 23].

In all the histopathological findings in our described cases, the existence of a corrugated parakeratotic layer of epithelial cells was emphasized, which is a basic feature of OKC. For this reason, we consider that histopathological analysis is imperative in setting the diagnosis of these cysts. In other words, the obligation of each surgeon is to perform a biopsy and send the sample to a pathologist for analysis. Given that the finding of parakeratosis is linked to a tendency to relapse, all our patients were warned of this possibility and advised that regular controls are crucial. The follow-up period of the described cases ranged from 1 year to 9 years. In the opinion of a large number of authorities, it usually occurs 2 years postoperatively, so OKC detected histopathologically should be monitored for at least 5 years postsurgery [2]. For Case #1 and Case #2, we can expect a good prognosis. Case #3 and Case #4 should continue to be controlled over the next several years. Of all the described cases, the authors of this paper find that the possibility of recurrence is most evident in Case #3 because of the existence of the so-called satellite cysts described in the histopathological findings.

Although OKCs are strictly diagnosed only by histological examination, there are some radiological and other features that can be of help in approaching the diagnosis, with an indication that these features are not pathognomonic for odontogenic keratocysts [18]. For example, the most common mandibular localization of OKC is the posterior part (angle and the ramus) [24–26] where the anterior section of the maxilla is the most common OKC localization in the upper jaw [27]. OKC can be also seen in the molar region of the maxilla [27]. Three of our described cases were localized in the maxilla, two in the anterior part of the maxilla and one in the region of an unerupted third molar. One case was localized in the posterior part of the mandibula.

The radiological appearance of OKC is unilocular and multilocular. The same appearance can be seen in other jaw lesions of odontogenic and nonodontogenic origin. This is important in differential diagnosis where OKC may be misdiagnosed as other odontogenic and nonodontogenic cysts and ameloblastoma [18]. Unilocular lesions in relation to impacted teeth can look like dentigerous cysts, which is seen most in young patients. For example, in our described cases (Case #1 and Case #2), OKCs were justifiably misdiagnosed as dentigerous cysts. Unilocular radiolucency located between the roots of the adjacent teeth can be mistaken for a radicular cyst (this was presented and described in Case #4).

OKCs, especially large ones, show a specific pattern of growth. Large mandibular OKCs grow in a mesiodistal direction along the length of the bone, with minimal buccolingual expansion. This type was also recognized in Case #3. An OKC located in the maxilla reveals expansion of the cortical bone, which can be seen as a bone deformity [18]. This characteristic was obvious in our Cases #1 and #4.

One of the consequences of the described growth pattern of OKC in the upper and lower jaws is that these cysts are usually asymptomatic for a long period of time. Patients usually seek help from doctors if deformities in the bones are noticed (Cases #1 and #4) or if spillage of cystic pus (Case #2) or a fistula is present (Case #3).

Of the other clinical features, OKC cystic fluid is also important. This content can be of different viscosities and colors, from straw yellow and thin to cheesy and thick. For Case #2 and Case #3, this content was dense in consistency and dirty yellow in color, and in Case #4 light yellow and murky. In Case #1, the content of the cyst was not a dominant symptom in the clinical findings. In addition to the question of classification, it is even more interesting to consider the therapeutic possibilities for OKC. There are two methods in the treatment of these lesions, one conservative and the other aggressive [9, 28–37]. The conservative method involves enucleation with or without curettage [38], decompression [39], and marsupialization [7]. Aggressive methods include peripheral ostectomy (with rotating instruments) [40], cryotherapy (with liquid nitrogen) [40], and application of Carnoy's solution [41]. All these methods have common goals: enucleation of the cyst and reducing the risk of recurrence and surgical morbidity. However, it is very difficult to monitor the therapeutic results in various studies, due to the small sample size, their retrospective nature, the deficiencies of the details described of the therapeutic procedures, and the variability of the control checkups [4, 17, 22, 42].

The reported recurrence rates for OKC vary from 5% to almost 70%, depending on the therapeutic procedure [43]. There are several theories explaining OKC recurrence, including the incomplete removal of the epithelial cyst lining and the growth of new cysts from small satellite cysts or odontogenic residues left behind during the operation [43]. Surgical factors are considered to have a significant impact on the probability of recurrence.

It is certain that an aggressive and radical approach to therapy will lead to the reduced possibility of recurrence, but the question arises: what if we have a young patient who is still in the phase of eruption of permanent teeth? By aggressive therapy, the integrity of the jaw bone would be violated and the procedure would practically mutilate the patient without any certainty as to whether there would be recurrence in general [7]. In the case of a ten-year-old patient (Case #1), we chose to enucleate the cyst, which was our only therapeutic choice, especially as the clinicians assumed it was a dentigerous cyst rather than a keratocyst, which was subsequently diagnosed histopathologically. When considering our Case #1, the question arises, would perhaps a better therapy option be to undertake marsupialization or decompression and thereby create the opportunity for spontaneous eruption of the retained canines?

Dong et al. conclude that children with such cysts and with permanent teeth still to erupt should be treated conservatively rather than aggressively, since aggressive treatment may have a negative effect on tooth development, eruption processes, and jaw development [17]. On the basis of the cases described by various authors, we believe that marsupialization followed by enucleation has the lowest recurrence rate in comparison with all conservative methods.

Pitak-Arnnop et al. consider that decompression and marsupialization are useful in some cases [2]. Both methods result in a significant reduction in cystic volume and a decrease in the possibility of injury to important anatomical structures, such as the inferior alveolar nerve; they allow biopsy and facilitate the subsequent complete removal of the cyst. These methods also reduce IL-1*α* and cytokeratin-10 that are thought to be related to cyst expansion. In addition, after decompression and marsupialization, the epithelial lining converts in a less aggressive form. However, both of these methods imply the longer duration of therapy, a greater number of interventions, and in particular the patient's cooperation. It is also possible that the biopsy sample that is taken during decompression and marsupialization is not representative [7].

Pitak-Arnnop et al. devised an algorithm in 2009 for the treatment of OKC and other cyst-like lesions. Thus, regardless of the histological types and localization of lesions, they favor enucleation in combination with adequate postoperative control [2]. This approach has been used for many years, not only for benign but also for aggressive lesions such as ameloblastoma [6, 13].

It is ideal to remove the cyst in one piece. If the cyst is located periapically or if access is difficult and if it is a recurrent cyst, peripheral ostectomy and/or curettage is recommended. After both approaches, histopathology is obligatory. If an odontogenic keratocyst is confirmed, this does not mean that the surgery should be repeated immediately because 25-50% of cases recur after a simple enucleation. Thus, a “Wait and see!” protocol should be applied [2].

Considering that in the differential diagnosis of OKC there are follicular-dentigerous cysts and ameloblastomas, in the therapeutic approach enucleation should not be avoided in order to remove the lesion in one piece. This would be our attitude, based on the doctrinal approach of many authors [39].

Many have tried to answer the question regarding which factors influence OKC recurrence.

It is still not known exactly where the epithelial islands and microcysts are located. They could be localized in a cyst capsule, in the overlying soft tissue, and/or in the bony bed of the cyst. Carnoy's solution and liquid nitrogen are recommended for epithelial island and microcyst elimination [2, 44]. It is considered that these agents significantly reduce the cyst recurrence rate [45].

As recent research has shown that most of the epithelial islands and/or microcysts are located in the mucosa that is directly in contact with the cyst, it is not necessary to remove and freeze the adjacent bone tissue. Moreover, peripheral ostectomy may disperse microcysts that are immersed in the bone and thus contribute to a higher risk of recurrence [7, 43].

Over the past few years, a great deal of attention has been dedicated to new procedures in the treatment of OKC in order to make it as successful and simple as possible, thereby improving the final outcome of the therapy. One of the therapeutic agents considered in this procedure is an antimetabolic drug, 5-fluorouracil, which is believed to induce apoptosis of the cells in a hepatocellular carcinoma. In addition, 5-fluorouracil has other domains of use, such as its topical application in the treatment of basocellular carcinoma and keratocystic odontogenic tumors. For 5-fluorouracil, it is considered to be more acceptable in the treatment of a keratocystic odontogenic tumor than Carnoy's solution, due to its availability, technical simplicity, shorter operating time, and equally successful, reduced morbidity of other tissues compared to Carnoy's solution. In order to use it, it is necessary to soak the gauze in a 5% solution of this drug, which is then applied to the residual bone cavity so that it may easily be found 24 hours after surgery. In contrast, Carnoy's solution requires a much longer period of time when it comes to its use, given the fact that it is necessary to comply with the many precautions that this product requires. In addition, with the use of 5-fluorouracil, no side effects of the topical application were noticed in the study carried out by Ledderhof and associates. However, systemic administration of 5-fluorouracil may lead to certain side effects, such as mucositis, agranulocytosis, neuropathy, nausea, vomiting, hypotension, exhaustion, and even death. Death is usually due to a lack of enzyme dihydropyrimidine dehydrogenase (DPD) that breaks down this drug resulting in systemic toxicity. Most commonly, this enzyme is missing in African woman with a frequency of 12%, which should be considered in the systemic administration of this drug [46].

In the last few years, studies have also demonstrated the success of cyclopamine, a plant alkaloid, which inhibits cell response to the SHH (sonic hedgehog) signal. Cyclopamine blocks the activation of the SHH signaling pathway, thereby proving effective in treating OKC. This therapeutic possibility has not yet been fully developed, due to the lack of data on its success, as well as the fact that it requires research and action on the molecular level [43].

In all our described cases, a conservative therapeutic approach was adopted in the form of cyst enucleation without the use of Carnoy's solution or any other additional substance. The reason for this approach is based on the fact that Case #1 and Case #2 were primarily considered to be dentigerous cysts, which was why enucleation was planned as the only therapeutic option. Since histopathology diagnosed OKC, the patients are regularly monitored. In a case of suspected relapse, reenucleation with the use of Carnoy's solution would be shortlisted. In Case #4, the patient mentioned a traumatic blow in the region of the upper frontal teeth where the cyst was localized. This time a radicular cyst of traumatic etiology was suspected where enucleation was also the first choice. The patient is still being monitored regularly. As in previous cases, reoperation in the form of enucleation using Carnoy's solution will only be considered in the case of recurrence. Of all these cases, Case #3 was first suspected of having an OKC, primarily due to the radiological characteristics, localization of the lesion, and the age of the patient (a large radiolucent lesion around the semi-impacted tooth 35 in a female patient aged 52). The authors want to emphasize that at the time of preparation for surgery, they were not in possession of Figure 8. The patient failed to show us a controversial orthopantomogram she had undergone at a surgical control station, showing semi-impacted teeth and radiolucency around them, which suggested that it was a cyst, most probably a keratocyst. Considering the presence of satellite cysts in the histopathological finding, we consider Case #3 to have the greatest possibility of recurrence in relation to the other cases described. The problem is that the patient stopped coming to regular checkups.

Although there are many therapeutic approaches for this type of cyst found in literature, there is still a lack of a universal therapeutic approach [9, 29, 47]. The reason for this is that each therapeutic modality has both advantages and disadvantages. For example, radical resection has the lowest recurrence rates [31, 32, 37, 48]; however, it leads to excessive morbidity (tooth and bone loss, facial asymmetry, secondary infection, and others) [31, 33]. A conservative approach is also a popular treatment option, especially after the reclassification from tumors to cysts (KCOT to OKC) [9]. The most widely used conservative approach is the combination of conservative therapeutic modalities (decompression and enucleation) in combination with additional techniques (peripheral ostectomy, use of Carnoy's solution, cryotherapy, and excision of the overlying mucosa) [9, 48, 49].

The advantages of a conservative approach include fewer morbidities than radical resection [48]. The disadvantages include rupture of the cyst capsule during cyst enucleation, which is associated with a higher recurrence rate [31]. Since there is no universal guideline for OKC treatment, whichever approach is used, it is necessary to eliminate the epithelial cyst remnants and satellite cysts, which are responsible for recurrence [31].

## 4. Conclusion

In terms of differential diagnosis, in the case of younger patients (children and adolescents), when considering a dentigerous cyst, one should refer to odontogenic keratocysts. Enucleation of OKC with regular follow-up proved to be an adequate therapeutic choice for the patients described in this paper. Only in the case of recurrence would other therapeutic options be considered, such as enucleation in combination with Carnoy's solution.
